# Supplementary material for: The Impact of A3AR Antagonism on the Differential Expression of Chemoresistance-Related Genes in Glioblastoma Stem-like Cells
Source: Pharmaceuticals (Basel). 2024 Apr 30;17(5):579. doi: 10.3390/ph17050579 (PMC11124321; doi:10.3390/ph17050579)
Supplement: Supplementary file 1 [file pharmaceuticals-17-00579-s001.zip › pharmaceuticals-2868326-supplementary.pdf]

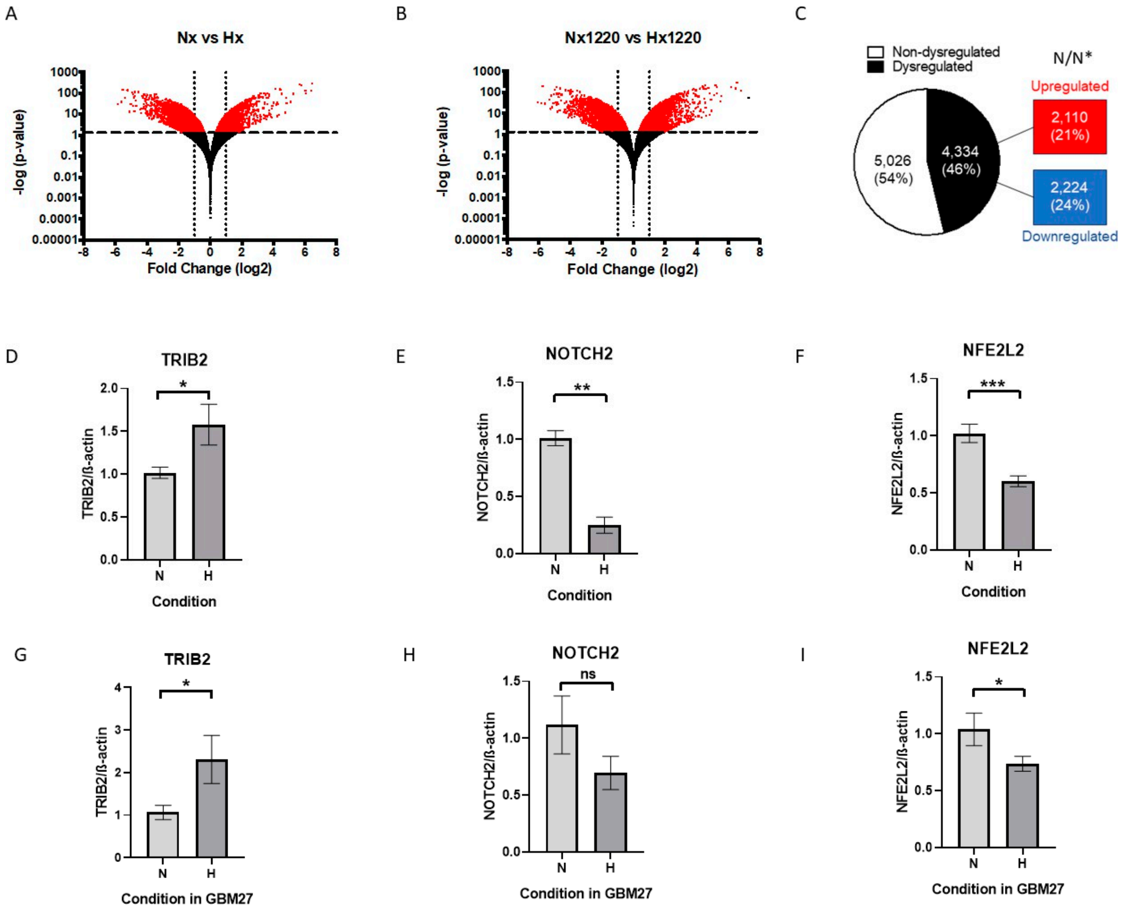

Figure S1: Volcano plots and validation of RNAseq. A) Volcano plot of genes differentially regulated by hypoxia compared to a normoxia condition in U87-GSC cells. B) Volcano plot of the genes differentially regulated by MRS1220 in hypoxia compared to the normoxia condition treated with MRS1220 in U87-GSC cells. C) Summary of genes differentially regulated by MRS1220 in a normoxia condition. D) Expression analysis of the TRIB2 gene performed with relative quantification by RTqPCR in U87MG-GSC in Normoxic and Hipoxic condition. E) Expression analysis of the NOTCH2 gene performed with relative quantification by RTqPCR. in U87MG-GSC in Normoxic and Hipoxic condition F) Expression analysis of the TRIB2 gene performed with relative quantification by RTqPCR in U87MG-GSC in Normoxic and Hipoxic condition. G) Expression analysis of the TRIB2 gene performed with relative quantification by RTqPCR in GBM27 in Normoxic and Hipoxic condition. H) Expression analysis of the NOTCH2 gene performed with relative quantification by RTqPCR. in GBM27 in Normoxic and Hipoxic condition I) Expression analysis of the TRIB2 gene performed with relative quantification by RTqPCR in GBM27 in Normoxic and Hipoxic condition. RTqPCR were performed in triplicate.

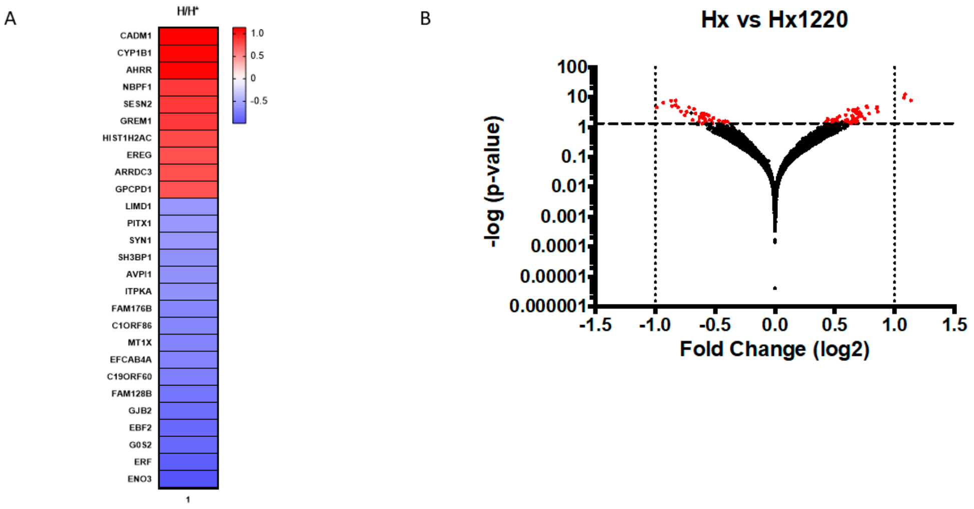

Figure S2: Heat Map and volcano plot Hipoxia vs Hipoxia MRS1220. A) A heat map of the hypoxia vs hypoxia condition with MRS1220 treatment shows the top 15 positively regulated genes and the top 15 negatively regulated genes. B) Volcano plot of genes differentially regulated by MRS1220 compared to a hypoxic condition in U87-GSC cells.

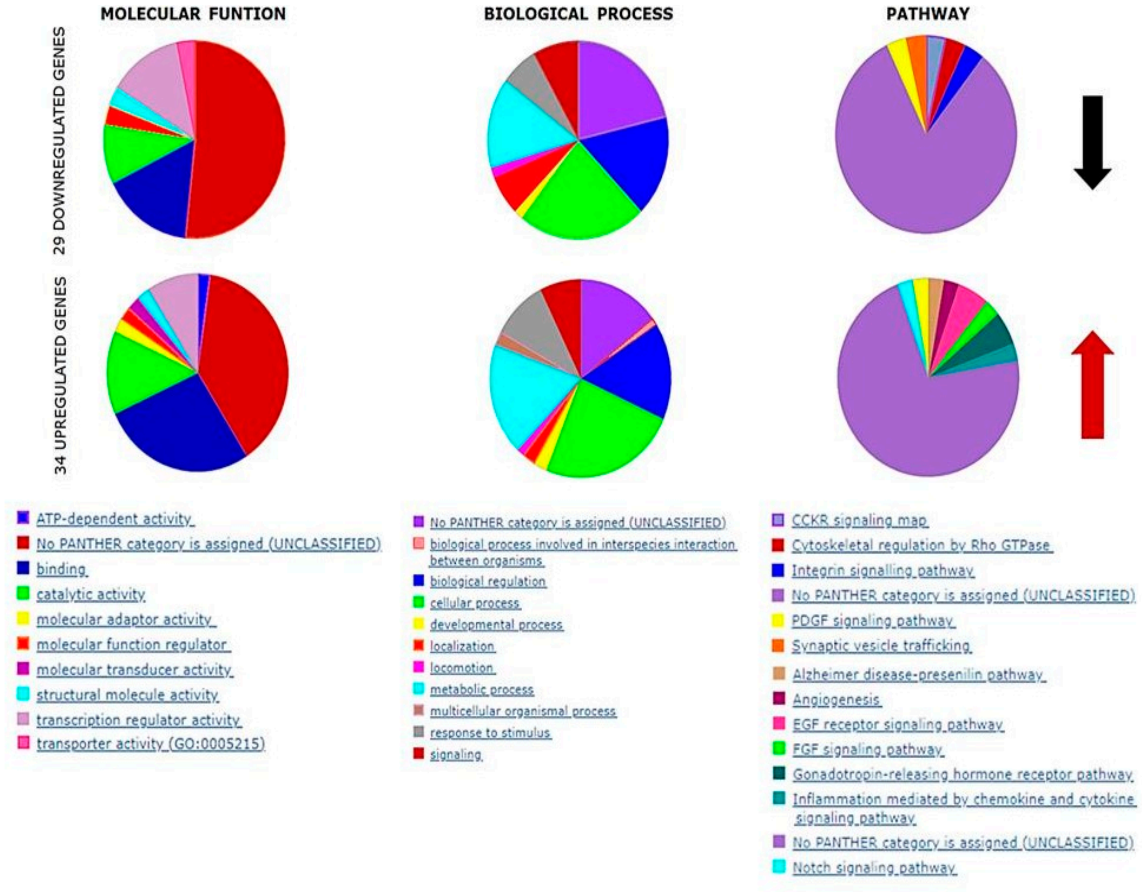

Figure S3: Molecular functions, biological processes, and pathways related to the down and upregulated genes after MRS1220 treatment in hypoxic conditions.

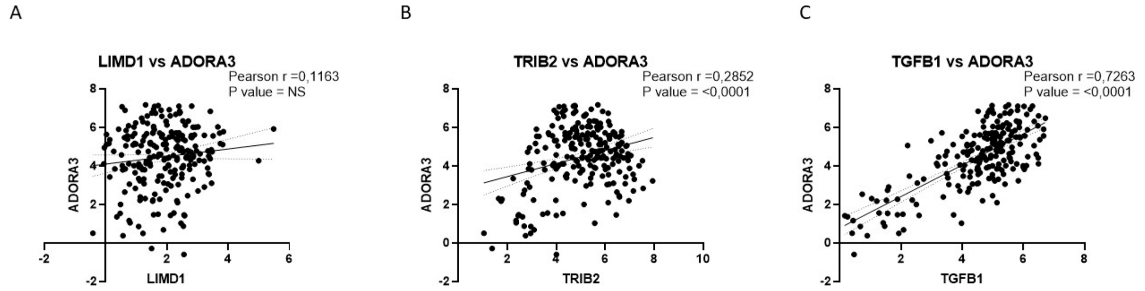

Figure S4: Person correlation analysis between LIMD1, TRIB2 and TGFB1 with the expression of the A3 receptor in glioblastoma. A) Person correlation of LIMD1 messenger expression with ADORA3. B) Person's correlation of TRIB2 messenger expression with ADORA3 C) Person's correlation of TGFB1 messenger expression with ADORA3. Person r and P values are shown in all correlation graphs. The data were obtained from the GlioVis CCGA. Data are presented as the mean ± standard deviation (SD); * $p < 0.05$; *** $p < 0.001$ and **** $p < 0.0001$
